# Exploring public sector physicians’ resilience, reactions and coping strategies in times of economic crisis; findings from a survey in Portugal’s capital city area

**DOI:** 10.1186/s12913-017-2151-1

**Published:** 2017-03-15

**Authors:** Giuliano Russo, Carlos André Pires, Julian Perelman, Luzia Gonçalves, Pedro Pita Barros

**Affiliations:** 1 UEI SPIB, Universidade Nova de Lisboa, Rua da Junqueira 100, Lisbon, Portugal; 2 Centre for Primary Care and Public Health, Queen Mary University of London, 58 Turner Street, Yvonne Carter building, London, E1 2AB UK; 3 Centre for Mathematics, University of Trás os Montes e Alto Douro, Vila Real, Portugal; 4 Escola Nacional de Saúde Pública, Universidade Nova de Lisboa, Lisbon, Portugal; 5 Centro de Estatística e Aplicações da Universidade de Lisboa, Lisbon, Portugal; 6 Nova Business School, Universidade Nova de Lisboa, Lisbon, Portugal

**Keywords:** Physicians and economic crisis, Health services Portugal, Physicians resilience and coping strategies, Health services economic crisis, Portugal’s healthcare system

## Abstract

**Background:**

Evidence is accumulating on the impact of the recent economic crisis on health and health systems across Europe. However, little is known about the effect this is having on physicians - a crucial resource for the delivery of healthcare services. This paper explores the adaptation to the crisis of public sector physicians and their ability to keep performing their functions, with the objective of gaining a better understanding of health workers’ resilience under deteriorating conditions.

**Methods:**

We conducted a survey among 484 public primary care and hospital physicians in Portugal’s capital city area and explored their perceptions of the crisis, adaptation and coping strategies. We used ordinal and logistic regression models to link changes in hours worked and intentions to migrate with physicians’ characteristics and specific answers.

**Results:**

We found little evidence of physicians changing their overall allocation of working time before and after the crisis, with their age, types of specialisation, valuation of job flexibility and independence significantly associated with changes in public sector hours between 2010 and 2015. Being divorced, not Portuguese, of younger age, and working a high number of hours per week, were found to increase the probability of physicians considering migration, the same as having a poor opinion of recent government health policies. On the other hand, enjoying their current working environment, not wanting to disrupt provision of service, and leisure time were found to protect against scaling down public sector hours or considering migration.

**Conclusions:**

Our work on Portuguese physicians contributes to the debate on health workers’ resilience, showing the value of understanding the influence of personal characteristics and opinions on their adaptation to changing circumstances, before designing policies to improve their working conditions and retention.

**Electronic supplementary material:**

The online version of this article (doi:10.1186/s12913-017-2151-1) contains supplementary material, which is available to authorized users.

## Background

The economic and financial crisis is believed to be taking its toll on the health of Europeans and on their health systems [1–3], although recent studies are emerging on health systems’ resilience in times of economic crisis [4–6]. Because of the decrease in consumers’ disposable income, raising unemployment, and controversial austerity measures introduced to ration demand and balance budgets, there are signs that population health status, demand for health care and health policies are beginning to be affected, particularly in Southern European countries like Greece [2, 7], Italy [8, 9], Spain [10–12] and Ireland [13]. Less is known of how health workers are reacting to the crisis, and whether they still guarantee the services needed, although some preliminary qualitative evidence is beginning to emerge [11, 14, 15]. Public sector physicians are particularly crucial, given the volatility of such a costly resource, and the pivotal role they play in the organisation of health services and in the definition of policies [16].

Physician resilience has been explored from different angles. The medical and psychological literature has long explored individuals’ resilience, particularly doctors’ burnout and their ability to resist stress and wearing workloads [17]. While ‘individual resilience’ typically describes the capacity of individuals to cope with adversity and continue functioning [18], ‘physician resilience’ has been defined as physicians’ ability to respond to stress induced by the medical profession so that professional objectives are achieved with minimal psychological and physical costs [19]. The importance of the strategies individuals adopt to cope with stressful and disrupting events has often been highlighted [20], and some authors maintain that some physicians are more resilient than others not only because of their personal characteristics, but also because of the different coping strategies they adopt [21]. As for health systems in economic crises, ‘resilience’ has been framed as people’s and institutions’ intrinsic ability to change and adapt to a lower level of resources to keep performing the same functions [22].

Economic theory predicts that with shrinking public salaries, physicians will reallocate time towards more profitable alternatives and away from the public sector to meet their target income, subject to the availability of private sector employment [23]. There is already evidence that some specific countries’ negative economic outlook is forcing nurses to leave [24]; evidence from Greece and Ireland shows that unemployment, job insecurity, income reduction and also a lack of opportunity for medical research are pushing young physicians to seek jobs abroad [25–27]. Recent studies from Spain show strong opposition to the austerity measures recently introduced to the health sector [11, 15].

In the Portuguese case, austerity measures started to be introduced in 2010 with substantial implications for its National Health System [28]. Although salaries were cut and income tax was increased, as well as salary bonuses eliminated across the board in the public sector, the health sector was specifically targeted for expenditure-saving and productivity-enhancing measures. Tertiary care hospital budgets were slashed, financial controls introduced for the most expensive tests and medical procedures, and a cap on personnel extra hours imposed; pay-for-performance contracts were reviewed with primary care Health Centres Groups (ACES). Amid concerns that such measures could reduce the breadth and depth of public healthcare services in a country where out of pocket expenditures still represent a sizable proportion of health expenditures [29], recent evidence links the crisis-related rise in unemployment with the decreased use of hospital services [30]. The present work aims at providing evidence on the adaptation of public sector physicians during an economic crisis, making a contribution to the debate on the impact of the crisis on physicians’ work in Europe, and on the drivers of physician migration. This should inform the government’s attempts to retain health professionals in the system and improve their conditions in the public sector, ultimately protecting access to affordable healthcare services in times of economic recession.

## Methods

We looked at changes in physicians’ *public sector working hours* between 2010 and 2015, and at *their intentions to migrate*, under the assumption that recent changes introduced by the crisis and related austerity measures would be somewhat reflected in these two proxies of physicians’ dedication and commitment to their job. Through multivariate analysis, we explored the association between these two dependent variables and physicians’ personal characteristics as well as their valuations of possible coping strategies and mitigating factors, as proxies for unobservable variables.

### Data collection

We surveyed a random sample of primary care and hospital specialists from three healthcare institutions in the Lisbon area in Portugal, and asked to report changes occurring before and after the economic crisis in terms of: (a) their perceptions of demand and supply of services in the public and private sectors; (b) their time allocation across professional activities; (c) strategies adopted to cope with the changes; and (d) mitigating factors.

Data were collected between January and July 2015 by a team of 15 interviewers. Survey interviews were conducted face-to-face following previous telephone contact. The three locations were selected to cover all the relevant types of medical specialisations. The study survey sample included a total of 484 physicians (72% of the physician population across the three locations). Physicians’ response rate was 90.98%, and no systematic difference was identified between the 48 physicians who refused to participate in the survey and those who did. Proportional stratified samples were obtained for gender, professional category, specialisation and institution (Additional file 1: Table S1).

### Data analysis

The survey questionnaire comprised 56 close-ended questions, and the resulting data set includes: (a) information on physicians’ personal characteristics; (b) variables related to their professional activity; (c) their stated intentions to migrate and valuation of factors known to mitigate the effects of stressful circumstances. The last variables resulted from questions presented in a Visual Analogue Scale ranging from 0 to 10 (Additional file 1: Table S2). The statistical analysis was performed using SPSS – version 22 and R program [31].

With the objective of analysing the association between changes in physicians’ public working hours and the above variables, we performed two ordinal regression models where the response variable, $Y$, was an ordinal variable with three categories. This variable represents the change in public working hours between 2010 and 2015: 1 = decreased, 2 = the same, and 3 = increased. The ordinal model can be expressed as:

$$ logit\left[ P\left( Y\le c\right)\right]= \ln \left(\frac{P\left( Y\le c\right)}{1- P\left( Y\le c\right)}\right)= \ln \left(\frac{P\left( Y\le c\right)}{P\left( Y> c\right)}\right) = {\upalpha}_c-\left({\beta}_1{X}_1+\dots +{\beta}_p{X}_p\right) $$

where $Y$ is the dependent variable, *c* = 1, 2 represents the first and second categories of the dependent variable, ${X}_1,\dots, {X}_p$ are the *p* explanatory variables, and ${\beta}_1,\dots, {\beta}_p$ the regression coefficients. The assumption of proportional odds was tested with the test of parallel lines implemented in SPSS [27]. Zero inflated models were also explored to gain a better understanding of differences in hours worked in public between 2010 and 2015, analyzing “zeros” and “count of non-zeros” [32].

To study the factors affecting the decision to migrate, two binary logistic regression models were performed, both with the intention to migrate as dependent variable (0 = No; 1 = Yes). In the first model, personal characteristics and variables related to the professional activity entered as explanatory variables, using the forward stepwise method. In the second model, the significant variables from the first model were entered as a block, and the variables related to the perceptions on current health policies and to the reasons why migrating is not a valid option were entered as a second block, using the forward stepwise method. Both models can be expressed as:

$$ logit\left(\pi \right)= \ln \left(\frac{\pi}{1-\pi}\right)=\upalpha +{\beta}_1{X}_1+\dots +{\beta}_p{X}_p $$

with $Y$ the dependent variable, $\pi$ the probability of thinking of migrating, ${X}_1,\dots, {X}_p$ the *p* explanatory variables, and ${\beta}_1,\dots, {\beta}_p$ the regression coefficients [33].

## Results

Physicians in our sample were predominantly of Portuguese nationality (97%), female (57%) and with a median age of 43 years (IQR: 31–57) (Table 3). There were more male physicians in the hospital sample than in the primary care institutions (53% in São José Hospital vs 24% in Cascais and 29% in Amadora); the former were also slightly younger, with a smaller proportion married. Considerably more hospital specialists engaged with private sector activities than their primary care counterparts (46% vs 27 and 24%). A higher proportion of medical residents were found in the hospital sample (34%) than in the health centre groups (Table 1).

**Table 1:** Sample characteristics, by healthcare unit

	Total (*N* = 484)	Healthcare units		
Characteristics		HSJ (*n* = 302)	ACES Cascais (*n* = 96)	ACES Amadora (*n* = 86)
Age (Median, IQR)	43.00 (31–57)	42.00 (30–55)	47.00 (33–58)	43.00 (32–58)
Gender (M) n(%)	208 (43.0%)	160 (53.0%)	23 (24.0%)	25 (29.1%)
Married (Y)	260 (53.7%)	155 (51.3%)	54 (56.3%)	51 (59.3%)
Nationality (Pt)	473 (97.7%)	298 (98.7%)	92 (95.8%)	83 (96.5%)
Other physician in family (Y)	246 (50.8%)	157 (52.0%)	42 (43.8%)	47 (54.7%)
Dependents (Y)	227 (46.9%)	139 (46.0%)	47 (49.0%)	41 (47.7%)
Years as Medical Doctor	18.45 (12.98)	18.04 (12.98)	19.60 (12.62)	18.59 (13.43)
Private sector practice (Y)	185 (38.3%)	138 (45.8%)	26 (27.1%)	21 (24.4%)
Junior medical residents	10 (2.1%)	10 (3.3%)	0 (0.0%)	0 (0.0%)
Senior medical residents	132 (27.3%)	92 (30.5%)	17 (17.7%)	23 (26.7%)
Clinicians (no spec.)	6 (1.2%)	1 (0.3%)	3 (3.1%)	2 (2.3%)
Auxiliary assistant	142 (29.3%)	80 (26.5%)	36 (37.5%)	26 (30.2%)
Graduate assistant	147 (30.4%)	87 (28.8%)	32 (33.3%)	28 (32.6%)
Senior graduate assistant	47 (9.7%)	32 (10.6%)	8 (8.3%)	7 (8.1%)
Total weekly working hours (2015): Mean (SD)	52.00 (13.07)	55.85 (14.22)	46.70 (8.55)	44.97 (6.73)
Public weekly working hours (2015): Mean (SD)	45.64 (11.26)	47.53 (13.09)	42.85 (6.61)	42.29 (5.94)
Weekly Leisure Time (2015) Mean (SD)	15.85 (12.12)	16.12 (12.82)	15.71 (11.05)	15.08 (10.75)
Total yearly earnings (2015) (1000*Euros): Mean (SD)	33,611 (21,020)	36,347 (24,539)	30,435 (13,591)	27,998 (11,415)

*IQR* interquartile range; Qualitative variables characterized as n(%)

Between public and private sector activities, hospital physicians declared working more hours per week in 2015 (median value of 56) than those from health centres (47 and 45, respectively). Total declared earnings were also considerably higher for hospital physicians (€36,350 per year in 2015) than their primary care peers (€30,440 and €28,000, respectively).

**Fig. 1:**
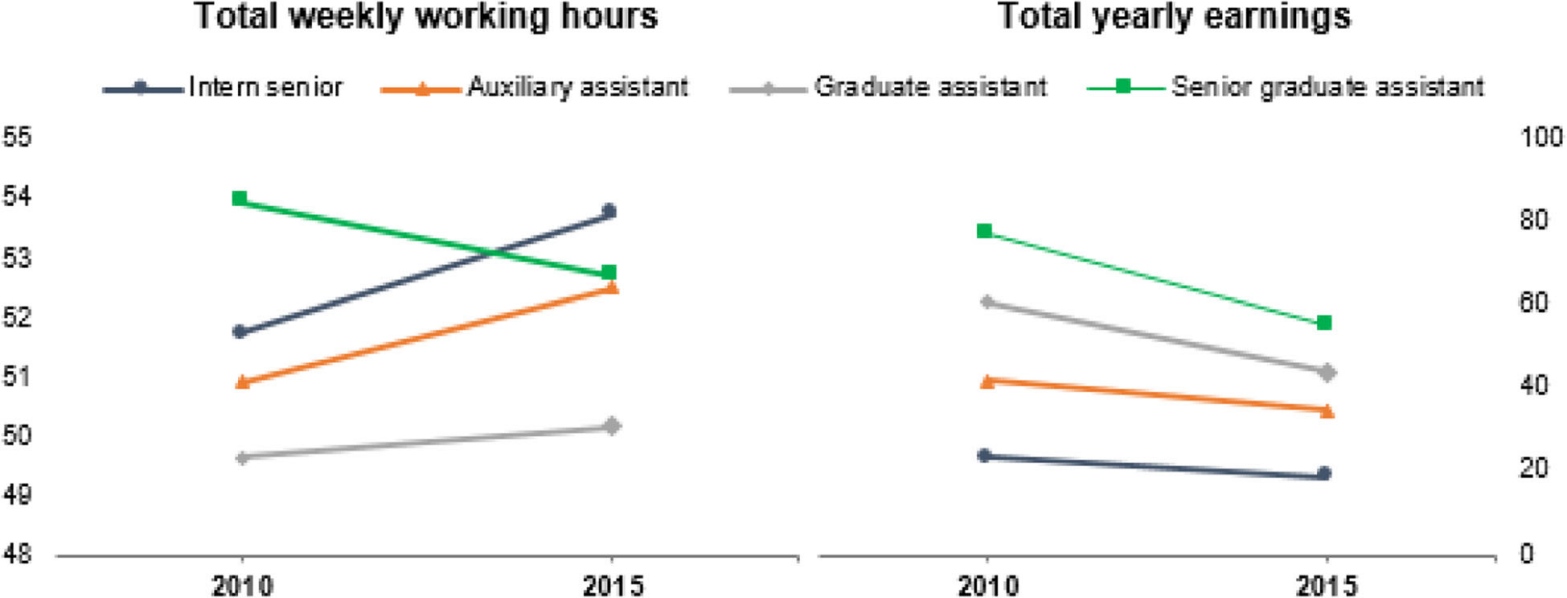
Working hours and earnings before and after the crisis, per type of physician (means)

Between 2010 and 2015, overall declared yearly earnings by the sampled physicians decreased by 35% (from €51,550 to €33,610), with the largest declared reduction accruing from their public sector earnings. The most senior cadres across the three institutions and the hospital specialists in particular reported the most substantial cuts (Fig. 1).

In the same period, with the exception of Senior Graduate Assistants, total working hours increased from 50.9 to 52.0 per week, with the increase attributable to extra hours worked in the public sector. Overall, we found no significant reduction of private sector earnings and working hours between 2010 and 2015 (Wilcoxon test: *p* = 0,867 and *p* = 0,436, respectively).

### Changes in public sector working hours

**Fig. 2:**
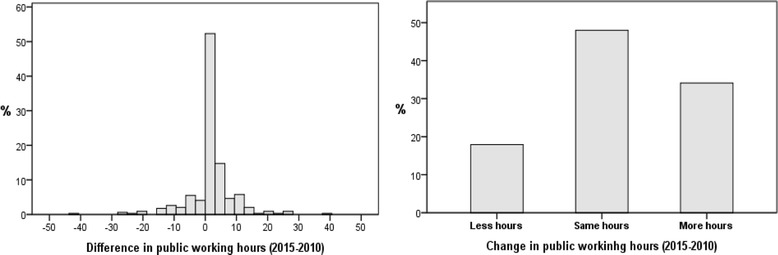
Constructed variable of differences in hours worked in public between 2010 and 2015

A variable was constructed measuring the 2010–2015 difference in hours worked in the public sector (Fig. 2, left-hand graph). Almost half the physicians (48%) declared working the same hours as in 2010. Such variable was categorised into three groups, namely: those physicians declaring working fewer hours in 2015 than in 2010; those declaring working the same hours; and those declaring working more (Fig. 2, right-hand graph).

Such categorised variable was used as a dependent variable for an ordinal regression model, estimating the association between physicians’ location, personal characteristics, their valuation of specific profession characteristics, and the 2010–2015 change in public sector working hours. In the first model (Model 1, Table 3 below), being a hospital specialist increased the probability of working fewer hours in comparison to the reference category (all other primary care specialist bar *USF Modelo B* ones) (*p* < 0.001). Age increases were associated with higher probability of working fewer hours (*p* = 0.001); additional hours worked in the private sector in 2015 (clinical and non-clinical) also had the same effect on public sector working time (*p* = 0.025).

In the second model (Model 2, Table 2), valuation on job flexibility and independence was associated with fewer hours worked in the public sector (*p* = 0.004). On the other hand, valuation given on the possibility of working extra hours in the public sector, enjoying their current working environment and not wanting to leave for fear of disrupting provision of services were associated with an increase in weekly hours worked in the public sector in 2015 (respectively, *p* = 0.079; 0.067 and 0.023). In this second model, being a physician from a USF *Modelo B* became a significant positive factor (*p* = 0.106).

**Table 2:** Estimates of the ordinal model for change in the number of public sector working hours (*N*=328)

Independent variables	Model 1		Model 2	
	B (se)	*p*	B (se)	*p*
Type of HCU				
Other (USF Modelo A + UCSP + USP)	–	–	–	–
Hospital	−1.041 (0.244)	<0.001	−0.910 (0.259)	<0.001
USF Modelo B (*n* = 16)	0.686 (0.580)	0.237	1.056 (0.654)	0.106
Age (years)	−0.031 (0.010)	0.001	−0.035 (0.010)	0.001
Private+other working hours 2015 (hours/week)	−0.024 (0.011)	0.025	−0.018 (0.012)	0.131
Valuation of job flexibility and independence	–	–	−0.112 (0.039)	0.004
Valuation of extra work in public (e.g.,. emergency shifts)	–	–	0.081 (0.046)	0.079
Valuation of enjoying of the current working environment	–	–	0.084 (0.046)	0.067
Not wanting to disrupt service in my job	–	–	0.077 (0.034)	0.023

The two models showed a good fit to the data $\left({\upchi}_{(414)}^2=433.16;\mathrm{p}=0.249\;\mathrm{and}\;{\upchi}_{(620)}^2=618.59;\mathrm{p}=0.509\right)$, but only explained 17 and 23% of the effect (Nagelkerke R2 = 0.168 and 0.227). The Zero-Inflated models reinforced the findings from the ordinal regression.

### Intentions to migrate

The logistic regression models found significant associations between physicians’ declared intention to migrate in 2015 and some of their personal characteristics, their allocation of time across activities, their answers to questions on current health policies, and their reasons given for not considering migration a valid option.

In our first model, covering exclusively personal characteristics and time allocation decisions, age showed a strong negative association with migration (*p*<0.001). Being female and having dependents also showed a negative association, while being divorced and a foreigner (not of Portuguese nationality) appeared to increase the probability of considering migration (*p*-values of 0.024 and <0.001, respectively). The weekly working hours in 2015 increased the intention to migrate (*p*=0.004), while additional leisure time (log) was associated with an opposite effect (Table 3).

**Table 3:** Estimates of the logistic model for intention to migrate (*N*=385)

Independent variables	Model 1		Model 2	
	B (se)	*p*	B (se)	*p*
Age	−0.069 (0.018)	<0.001	−0.047 (0.021)	0.025
Gender (Female)	−0.723 (0.321)	0.024	−0.310 (0.383)	0.419
Civil status (Married/partnership)				
Single	−0.130 (0.431)	0.762	0.182 (0.517)	0.724
Divorced	2.719 (0.557)	< 0.001	2.651 (0.656)	< 0.001
Nationality (Other)	1.799 (0.701)	0.010	2.133 (0.846)	0.012
Dependents (Yes)	−1.081 (0.443)	0.015	−0.441 (0.528)	0.404
Working hours 2015 (hours/week)	0.030 (0.011)	0.004	0.019 (0.012)	0.124
Leisure time 2015 (log (hours/week))	−0.482 (0.209)	0.021	−0.440 (0.252)	0.081
Health policies that damage public sector	–	–	0.402 (0.146)	0.006
Brain Drain	–	–	0.308 (0.117)	0.009
Enjoyment of the current working environment	–	–	−0.196 (0.068)	0.004
Current conditions of my current job	–	–	−0.153 (0.091)	0.092
Difficulty of finding a job abroad	–	–	−0.155 (0.081)	0.055
Family ties in Portugal	–	–	−0.126 (0.052)	0.015

In the second logistic regression model (Model 2, Table 3), valuations on thinking that recent policies and the current brain drain have damaged the sector were associated with considering leaving the country in the near future (*p*-values of 0.006 and 0.009, respectively). Valuations of physicians’ current working environment and of their remuneration conditions in comparison to other jobs in Portugal were found to decrease the probability of considering migration (*p*-values of 0.004 and 0.092, respectively). Also, valuation for family ties in Portugal (*p*=0.015) and considering it challenging to look for a job abroad (*p*=0.055) were factors associated with a decreased probability of considering migration, although this latter only at a 10% confidence level.

Both models fitted to the data (Hosmer and Lemeshow test: ${\boldsymbol{\chi}}_{(8)}^2=11.61; p=0.170\;\mathrm{and}\;{\boldsymbol{\chi}}_{(8)}^2=8.50; p=386$), and a good explanatory power, with area under ROC curves of 0.788 and 0.879, respectively. The Nagelkerke R^2^ was 0.240 for the first model end 0.415 for the second.

## Discussion

The public sector physicians in our sample displayed little reaction to crisis and related austerity measures in terms of reduction of public hours and intention to leave. A limited proportion of physicians declared their intention to leave the country in the short term, with specific personal characteristics and valuations of job features associated with such intentions. We did not find evidence of a shift in physicians’ time allocation towards private work, and this was unexpected, given physicians’ own perceptions of the private sector’s recent growth following the crisis. This could be explained either by the underrepresentation of private sector workers in our sample, or by the country’s private sector inability to provide job opportunities for all the physicians willing to engage with it. Despite its recent expansion, private medical employment in Portugal may still be an option only for selected physicians - the most senior ones, and for specific specialties - with junior doctors all but cut-off from real opportunities [34]. The implication of these findings for physician income behaviour theory would be that, although physicians may be rationally inclined to select the mix of public and private work that maximises their income, local market conditions would end up playing a larger than expected role in their allocation decisions across professional activities, which resonates with the arguments already put forward by some economics scholars [34, 35].

Our findings on the relation between work and leisure time, intentions to leave, and physicians’ appreciation of the characteristics of their job in Portugal suggest that some physicians may have decided to stay in their current employment, but to work less intensely in response to the deteriorating conditions. This would be consistent with labour economics theory on the income-leisure trade-off, as well as with the literature on physicians’ preferences for pecuniary and non-pecuniary job characteristics [36]. As the medical profession in Portugal still enjoys a privileged status and guarantees comparatively better remuneration even despite the recent cuts, it is easy to see why many physicians still consider acceptable their current terms of employment, and would be reluctant to seek riskier alternatives. It will be critical for policy purposes to understand better their demand’s wage elasticity, and identify a threshold for those combinations of labour and remuneration below which physicians may start leaving the public sector.

This paper adds to the growing body of knowledge on the impact of economic crisis on health and health systems, particularly in Southern European countries [2, 8, 11, 14]. It suggests that its effects on physicians may be more nuanced than originally thought because of the inherent resilience of the profession and of the wider health system. To some extent, our work lends support to existing theories of individual and professional resilience [19, 20] showing that physicians with specific characteristics (e.g., being married, having a strong personal and professional network with the country) build intrinsic resistance in the face of deteriorating conditions and increased workload. Rather than pointing towards a clear-cut reaction or adaptation strategy to the crisis, our findings are suggestive of piecemeal, differentiated adjustments that physicians in Portugal may be adopting to cope with changing circumstances, depending on their seniority and place of practice [14].

The findings above will have to be taken with a degree of caution, as our study is affected by limitations in terms of our approach to exploring resilience to the crisis, the sample’s representativeness, our use of physicians’ valuations as proxies for unobservable variables, and physicians’ responses on their own time allocations and earnings. We assumed any change in hours worked between 2010 and 2015 and intentions to migrate to be related to the crisis, but we cannot exclude the effect of confounding factors such as changes in individual physicians’ personal circumstances. We carried out our study in the Greater Lisbon area, which concentrates a large share of Portugal’s population and services, and may not be representative of the country in terms of physician specialties, workload, and opportunities for private sector employment. Ours was also a sample of physicians who decided (or had no alterantive but) to stay in the public sector; as a consequence, our findings may not be representative of the country’s general physician population. Finally, we asked physicians to state their earnings and allocation of time to activities before and after the crisis; such an exercise of recollection proved to be a difficult task for some interviewees, and we are aware it may have introduced a recall bias [37].

## Conclusions

We conducted a survey among public sector physicians in Portugal’s capital city area, and looked at how changes in allocation of their time across professional activities and intention to migrate were associated with personal characteristics and valuations of coping strategies and mitigating factors.

This study adds to the existing body of work on the impact of the economic crisis on Europe’s health systems and health professionals; although the physicians in our sample displayed overall little reaction in terms of public hours workload and intention to leave, we showed changes in the former to be associated with specific personal characteristics, place of work, and possible coping strategies. Some personal characteristics were found to have a negative effect on physicians’ intentions to migrate, but positive attitudes towards current employment and high valuations for family ties and for lifestyle in Portugal showed a negative association with migration intentions.

Despite the limitations, our findings further the debate on individual resilience and physicians' coping strategies in times of economic slowdowns, and on their terms of engagement with the labour market. This work shows the importance of understanding health workers’ reactions and adaptations to changing circumstances before designing policies to improve their work conditions and retention.

## Additional file

Additional file 1: Table S1. Survey sample, by type of institution and physician category. **Table S2.** Variable list and description. (DOC 14 kb)
