# Supplementary material for: Exploring public sector physicians’ resilience, reactions and coping strategies in times of economic crisis; findings from a survey in Portugal’s capital city area
Source: BMC Health Serv Res. 2017 Mar 15;17:207. doi: 10.1186/s12913-017-2151-1 (PMC5353948; doi:10.1186/s12913-017-2151-1)
Supplement: Additional file 1: Table S1. — Survey sample, by type of institution and physician category. Table S2. Variable list and description. (DOC 14 kb) [file 12913_2017_2151_MOESM1_ESM.doc]

Additional file 1

Table S1: Survey sample, by type of institution and physician category

| Physician category | São José Hospital | | ACES Cascais | | ACES Amadora | | Total | |
| --- | --- | --- | --- | --- | --- | --- | --- | --- |
|  | Sample | Universe | Sample | Universe | Sample | Universe | Sample | Universe |
| Senior graduate assistant | 32 | 32 | 8 | 9 | 7 | 7 | 47 | 48 |
| Graduate assistant | 87 | 116 | 32 | 43 | 28 | 40 | 147 | 199 |
| Assistant physician | 80 | 113 | 36 | 39 | 26 | 26 | 142 | 178 |
| General clinical practitioner | 1 | 2 | 3 | 6 | 2 | 4 | 6 | 12 |
| Senior medical residents | 92 | 144 | 17 | 31 | 23 | 39 | 132 | 214 |
| Junior medical residents | 10 | 24 | 0 | 0 | 0 | 0 | 10 | 24 |
| Total | 302 | 431 | 96 | 128 | 86 | 116 | 484 | 675 |

Table S2: Variable list and description

| **Variable** | **Description** |
| --- | --- |
| Healthcare unit | Public healthcare unit where the physician works: São José Hospital, ACES Cascais, or ACES Amadora. |
| Type of healthcare unit | Type of healthcare unit: hospital, USF Mod. B, or other (USF Mod. B, UCSP, USP) |
| Age | Age in years |
| Gender | 0=Male; 1=Female |
| Marital status | Married‎/partnership, Single, Divorced, Widowed |
| Nationality | Whether the physician is Portuguese or has another nationality: 0=Portuguese; 1=Other |
| Dependents | Whether the physician has dependents (children or other): 0=No; 1=Yes |
| Other physician in family | Whether the physician has another physician in family: 0=No; 1=Yes |
| Years as MD | Years working as a Medical Doctor |
| Specialty | Whether the physician has a specialization in medicine: 0=No; 1=Yes |
| Seniority | Professional category: Junior intern, Senior intern, General clinical practitioner, Assistant physician, Graduate assistant, or Senior graduate assistant |
| Private sector practice | Whether the physician works in the private sector: 0=No; 1=Yes |
| Other activities | Whether the physician has non-clinical professional activities: 0=No; 1=Yes |
| Private working hours | Weekly working hours in private sector (private + other activities) in 2010 and 2015 |
| Total working hours | Weekly working hours in public sector in 2010 and 2015 |
| Total yearly earnings | Total yearly earnings (public + private + other activities) in 1000*Euros in 2010 and 2015 |
| Leisure time | Weekly leisure time (hours) in 2010 and 2015 |
| Intention to migrate | “Are you considering migrating in the next 2 years?”: 0=No; 1=Yes |
| Factors that may have influenced the public sector in the last 5 years | “To what degree may the following factors have influenced the public sector in the last five years?” (Deteriorating conditions, Lack of motivation, Competition from private sector, PHC reform, Hospital reference reform, Change in patient case-mix, Brain-drain, Health policies that damage public sector).  Measured in a Visual Analogue Scale from 0 (minimum) to 10 (maximum). |
| Factors that helped ease the crisis impact | “In the case of feeling affected by the crisis, to what degree have the following factors helped ease its impact?” (Enjoying medical profession, Job's flexibility and independence, Extra work in public (e.g., emergency shifts), Exclusivity contract, Comparatively better conditions in my medical profession, Opportunity to work in private too, Possibility for early retirement, Partner's income, Financial support from the extended family, Return from physical assets)  Measured in a Visual Analogue Scale from 0 (minimum) to 10 (maximum). |
| Reasons to stay in current job | “To what degree do the following reasons lead you to stay in your current job?” (Enjoyment of the current medical profession, Enjoyment of the current working environment, Current conditions of my current job, Not wanting to disrupt service in my job, Appreciation for Portugal, Feeling not being the right age to migrate, Family ties in Portugal, Difficulty of finding a job abroad, Not speaking another language)  Measured in a Visual Analogue Scale from 0 (minimum) to 10 (maximum). |
